# Exposure of neonates to Respiratory Syncytial Virus is critical in determining subsequent airway response in adults

**DOI:** 10.1186/1465-9921-7-107

**Published:** 2006-08-07

**Authors:** Dahui You, David Becnel, Kai Wang, Michael Ripple, Melissa Daly, Stephania A Cormier

**Affiliations:** 1 Louisiana State University, Department of Biological Sciences, Baton Rouge, USA

## Abstract

**Background:**

Respiratory syncytial virus (RSV) is the most common cause of acute bronchiolitis in infants and the elderly. Furthermore, epidemiological data suggest that RSV infection during infancy is a potent trigger of subsequent wheeze and asthma development. However, the mechanism by which RSV contributes to asthma is complex and remains largely unknown. A recent study indicates that the age of initial RSV infection is a key factor in determining airway response to RSV rechallenge. We hypothesized that severe RSV infection during neonatal development significantly alters lung structure and the pulmonary immune micro-environment; and thus, neonatal RSV infection is crucial in the development of or predisposition to allergic inflammatory diseases such as asthma.

**Methods:**

To investigate this hypothesis the present study was conducted in a neonatal mouse model of RSV-induced pulmonary inflammation and airway dysfunction. Seven-day-old mice were infected with RSV (2 × 10^5 ^TCID_50_/g body weight) and allowed to mature to adulthood. To determine if neonatal RSV infection predisposed adult animals to enhanced pathophysiological responses to allergens, these mice were then sensitized and challenged with ovalbumin. Various endpoints including lung function, histopathology, cytokine production, and cellularity in bronchoalveolar lavage were examined.

**Results:**

RSV infection in neonates alone led to inflammatory airway disease characterized by airway hyperreactivity, peribronchial and perivascular inflammation, and subepithelial fibrosis in adults. If early RSV infection was followed by allergen exposure, this pulmonary phenotype was exacerbated. The initial response to neonatal RSV infection resulted in increased TNF-α levels in bronchoalveolar lavage. Interestingly, increased levels of IL-13 and mucus hyperproduction were observed almost three months after the initial infection with RSV.

**Conclusion:**

Neonatal RSV exposure results in long term pulmonary inflammation and exacerbates allergic airways disease. The early increase in TNF-α in the bronchoalveolar lavage implicates this inflammatory cytokine in orchestrating these events. Finally, the data presented emphasize IL-13 and TNF-α as potential therapeutic targets for treating RSV induced-asthma.

## Background

Respiratory syncytial virus (RSV) is the most common cause of upper and subsequent lower respiratory tract infection in children and the elderly and is most severe in children between the ages of 8 and 30 weeks [1]. Severe RSV infection occurs in 1–2% of the cases and may result in acute bronchiolitis that requires mechanical ventilation. [2].

Several studies have suggested that severe RSV lower respiratory tract infection in infancy may induce later development of asthma [3-8]. In 2000, Sigur and colleagues reported that RSV infection in infancy, severe enough to require hospitalization, was associated with asthma and allergy in children up to age 7 [9]. More recently, they reported that the relationship between severe RSV bronchiolitis in infancy and later development of asthma and allergy sensitization are still observed in children up to age 13. [10]. In murine models, numerous studies have been carried out and controversial results have been reported. While some studies propose that previous RSV infection enhances allergen sensitization and exacerbates asthma, others believe that RSV infection in infancy protects against later allergic sensitization [11]. This controversy is now thought to arise from the age of initial RSV infection and the different protocols used for RSV infection and allergen sensitization and challenge. In two independent experiments, researchers suggested that the timing of primary RSV infection is significant for predicting disease outcome. Culley and colleagues reported that if neonatal mice (1 d or 7 d of age) were infected with RSV and rechallenged at 12 wks of age, inflammatory cell recruitment was enhanced (including Th2 cells and eosinophils) [12]. Their data suggested that neonatal exposure to RSV exacerbated subsequent disease upon rechallenge in the adult. [12]. More recently, Dakhama and colleagues infected 1-wk old or 3-wk old mice with RSV, and they found that both primary infections led to increased lung resistance, mucus hyperproduction and inflammation along with increased lymphocytes in the lung. [13]. Furthermore, they demonstrated that the BAL fluid from mice infected with RSV at 1 wk of age had reduced levels of IFN-γ and elevated levels of IL-13 compared to mice initially infected at 3 wks of age. This data indicated that a Th2 polarized response occurred in mice infected with RSV at 1 wk of age, while a Th1-biased response occurred in the mice infected with RSV at 3 wks of age. When rechallenged with RSV 5 weeks later (i.e., infection at 1 wk and reinfection at 6 wks of age), these mice displayed exacerbated disease as indicated by enhanced pulmonary resistance, mucus hyperproduction, eosinophilia, and elevated levels of Th2 cytokines. In contrast, initial infection of mice at 3 wks of age resulted in protection upon RSV rechallenge as indicated by the abolished lung resistance and enhanced viral clearance at rechallenge [13]. These experiments indicate that age at initial RSV infection influences the subsequent immune and physiologic response upon re-exposure to RSV.

We hypothesized that neonatal exposure to RSV plays a critical role in the pathophysiological response to subsequent allergen exposure and the development of allergic asthma. To investigate whether neonatal exposure to RSV influences airway hyperreactivity (AHR), mice were infected with RSV at 1 wk of age. At 6 wks of age, these mice were then sensitized and challenged with ovalbumin (Ova). Pulmonary function, histopathology, and bronchoalveolar lavage fluid (BALF) cellularity and cytokine production were examined 24 hours after the last Ova challenge. The results were compared to sham infected and/or saline exposed mice and demonstrated that neonatal exposure to RSV resulted in elevated and prolonged AHR, chronic pulmonary inflammation, and subepithelial fibrosis in adult mice. These pathophysiological endpoints were most severe when neonatal RSV infection was followed by allergen sensitization and challenge. These studies demonstrate the ability of neonatal exposures to chronically alter lung function and the importance of preventing RSV exposure during infancy.

## Methods

**Figure 1:**
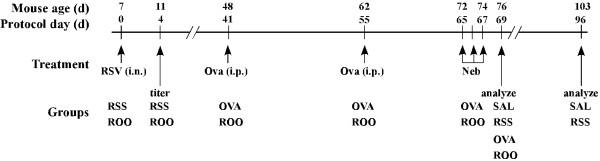
**Schematic of study protocol and exposure groups**. Neonatal mice (7 days of age) were infected with RSV (2 × 10^5 ^TCID50/g body weight; RSS, ROO groups). On protocol days 41 and 55, mice were injected i.p. with ovalbumin complexed to Imject Alum (OVA and ROO groups) or with isotonic saline (SAL and RSS groups). These mice were then exposed to aerosolized ovalbumin or saline for 20 minutes on protocol days 65, 66, and 67. n = 8/group.

The study protocol is outlined in Figure 1. Mice were divided into four groups. RSS mice were infected with RSV and mock-sensitized and challenged. OVA mice were mock-infected then Ova sensitized and challenged. ROO mice were infected with RSV and then Ova-sensitized and challenged. As the control group, SAL mice were mock infected, mock-sensitized and challenged. Data were collected on protocol days 0, 4, 69 and 96.

### Mice

BALB/c breeders were purchased from the Division of Laboratory Animal Medicine (School of Veterinary Medicine, Louisiana State University) and seven day old pups were obtained by time-mating. All mice were maintained in ventilated micro-isolator cages housed in a specific pathogen-free animal facility. Sentinel mice within this animal colony were negative for antibodies to viral and other known mouse pathogens. All animal protocols were prepared in accordance with the Guide for the Care and Use of Laboratory Animals (National Research Council, 1996) and approved by the Institutional Animal Care and Use Committee at Louisiana State University.

### Infection of mice with RSV and assessment of viral titers

Seven day old mice (protocol day 0; Figure 1) were infected intranasally (i.n.) with 2 × 10^5 ^TCID_50 _RSV/g body weight (RSV A-2, Advanced Biotechnologies Incorporated, Columbia, MD) or culture media alone (OVA group). Four days post-infection, viral titer of whole lung homogenates was determined using the TCID_50 _method of Spearman-Kärber [14,15].

### Ovalbumin sensitization and challenge

Mice were sensitized and challenged with chicken ovalbumin (grade V; Sigma, St. Louis, MO) as previously described [16]. Mice were intraperitoneally (i.p.) injected with 20 μg Ova complexed with 2 mg Imject Alum (Al [OH]3/Mg [OH]2; Pierce, Rockford, IL) or Sal (RSS and SAL group) on protocol days 41 and 55 (Figure 1) and subsequently challenged with aerosolized 1% Ova (in isotonic Sal) or Sal (RSS and SAL group) using an ultrasonic nebulizer (PariNeb Pro Nebulizer) on protocol days 65–67 (Figure 1).

### Assessment of pulmonary function

#### A) Lung resistance in response to methacholine

On protocol day 69, lung resistance to increasing doses of methacholine (MeCh, Sigma; 0, 6.25, 12.5, and 25 mg/ml in isotonic saline) was assessed using the forced oscillation technique as previously described [16]. Anesthetized animals were mechanically ventilated with a tidal volume of 10 ml/kg and a frequency of 2.5 Hz using a computer-controlled piston ventilator (FlexiVent, SCIREQ; Montreal, Canada). Resistance data were collected using single compartment model and plotted as the percent change from the 0 mg/ml MeCh dose.

#### B) Airway hyperreactivity in response to methacholine

Airway hyperresponsiveness to MeCh (Sigma; 0, 6.25, 12.5, 25 and 50 mg/ml in isotonic saline) was assessed by whole body plethysmography (Buxco Electronics, Troy, NY and EMKA Technologies, Falls Church, VA) as described previously [16]. Mice were exposed to aerosolized MeCh for 3 minutes at each dose and enhanced pause (Penh) was recorded for 3 minutes and averaged for each dose. Penh data were plotted as percent change from saline per dose.

### Determination of Bbronchoalveolar Lavage Fluid cellularity

On protocol day 69, bronchoalveolar lavage fluid (BALF) was isolated in 1 ml of PBS containing 2% heat-inactivated FBS. Total BAL cellularity was determined with the use of a hemocytometer. Cytospin slides were fixed and stained using the Diff-Quick kit (IMEB, Chicago, IL), and differential cell counts by two unbiased observers were obtained using standard morphological criteria to classify individual leukocyte populations. Three mice from each group were used for these analyses, and 200–300 cells were counted per cytospin preparation.

### Pulmonary histopathology

On protocol day 69, lungs were inflated with 1 ml of 4% paraformaldehyde. The lungs were then excised and fixed in 4% paraformaldehyde for 24 hours at 4°C. These tissues were then embedded in paraffin, cut in 4 μm frontal sections and stained with hematoxylin and eosin (H&E), periodic acid-Schiff (PAS) to show mucus production in airway goblet cells, or Masson's trichrome (MT) to indicate airway collagen deposition. Scores were assigned to these histological endpoints by two independent observers and were recorded on a scale of 0–3.

### Cytokine level in BAL fluid

IL-2, IL-4, IL-5, IFN-γ, and TNF-α levels in the BALF were examined using the Mouse Th1/Th2 Cytokine Cytometric Bead Array Kit (BD Biosciences, San Diego, CA) as per the manufacturer's instructions. Data were acquired with a BD FACScan™ flow cytometer. Data analyses were performed using the BD Cytometric Bead Array Software to generate standard curves for each cytokine and to determine sample cytokine levels. IL-13 levels in BALF were determined using the Mouse IL-13 ELISA Ready-SET-Go kit (eBioscience, San Diego, CA). The sensitivity for each cytokine is as follows: 5.0 pg/ml for IL-2, IL-4 and IL-5; 2.5 pg/ml for IFN-γ, 6.3 pg/ml for TNF-α and 30 pg/ml for IL-13.

### Statistical analysis

Data are presented as mean ± SEM and were obtained from experiments with n = 8 for whole body plethysmography analysis of AHR, n = 4–5 for invasive measurements of pulmonary mechanics, and n = 3 for BAL cellularity, cytokine assays, pulmonary viral titers and histology. For AHR, lung resistance and BAL cellularity, differences between groups were evaluated by means of two-way ANOVA using GraphPad Prism (GraphPad Software Inc, San Diago, CA). Bonferroni post-tests were performed to compare between pairs of groups. A one-way ANOVA was used to compare the mean cytokine levels among the various groups followed by the Tukey-Kramer multiple comparisons tests for significance between the groups. This was repeated for each individual cytokine. Differences between means were considered significant when p < 0.05.

## Results

### Reduced pulmonary function was observed in mice infected with RSV as neonates

To determine if neonatal exposure to RSV is sufficient to induce long-term pulmonary dysfunction, mice were infected with RSV (2 × 10^5 ^TCID/g body weight) at seven days of age and then allowed to mature to adults (Figure 1). Four days post-infection, we assessed pulmonary viral titers for Sham and RSV infected mice. As expected, mice from the Sham infected groups displayed no evidence of viral replication. The mean viral titer in the lungs of neonatal mice exposed to RSV alone (RSS and ROO) was 2.67 ± 1.29 × 10^6 ^TCID_50_/g lung tissue.

**Figure 2:**
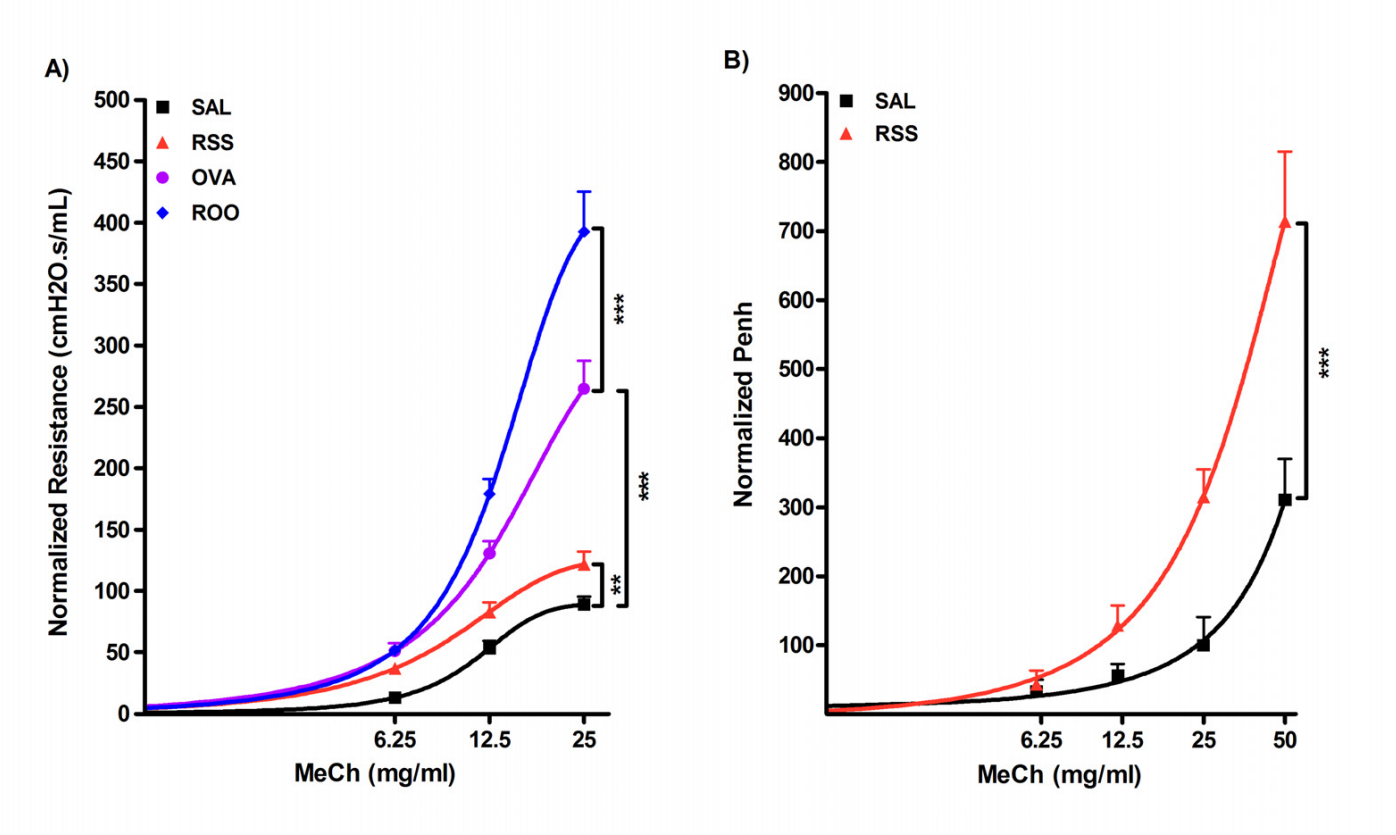
**Airway hyperreactivity in RSV and/or Ova exposed mice**. Mice were infected with RSV as neonates (RSS) and exposed to Ova 5 wks later (ROO). Controls were sham infected and exposed to Saline (SAL) or Ova (OVA). **A**. Lung resistance of each group is plotted as a function of increasing doses of inhaled MeCh, using single compartment model. **B**. Penh is plotted as a function of increasing dose of inhaled MeCh (0 to 50 mg/ml). Data were collected on protocol days 69 (A) or 96 (B), normalized to saline airway responses as described in methods, and expressed as mean ± SEM. n = 4–5/group. ***p < 0.001; **p < 0.01.

After maturation, subsets of mice were then sensitized (protocol days 41 and 55) and challenged (protocol days 65, 66, and 67) with ovalbumin – OVA and ROO groups. All other mice received saline – RSS and SAL groups. AHR was assessed on protocol day 69 and 96 (2.5 and 3.5 months after infection). We observed no significant difference in baseline airway resistance between the RSV infected (mean ± SEM; 0.52 ± 0.01 cm H_2_O.s/mL) or sham infected mice (0.64 ± 0.06). For ease of comparison among all groups, lung resistance data was normalized by plotting the percentage difference from baseline (percent difference = 100 * ((value-baseline)/baseline). Lung resistance in response to increasing concentrations of inhaled MeCh was significantly greater in RSV infected neonates (RSS; 121.79 ± 10.20) than control mice (SAL; 89.05 ± 6.51) at 25 mg/ml MeCh (1.4 times, p < 0.01) (Figure 2A). Neonatal RSV infection followed by exposure to Ova (ROO) resulted in the highest lung resistance among the four groups (392.50 ± 32.84). This increased airway resistance was greater than could be accounted for by Ova exposure alone (OVA; 264.63 ± 23.01). Thus, neonatal exposure to RSV infection appears to predispose adults to the development of airway responsiveness to subsequent allergen exposure.

To investigate the long-term influence of neonatal exposure to RSV, AHR was assessed by whole body plethysmography on protocol day 96 (103 days of age). Penh data was normalized and the percentage difference from baseline was plotted. Mice in the RSS group continued to demonstrate reduced airway function 3 months post-infection as evidenced by a significantly increased Penh compared to SAL mice at 25 and 50 mg/ml MeCh (314.30 ± 40.40 vs 100.00 ± 40.80 at 25 mg/ml; 714.30 ± 101.00 vs 311.10 ± 58.80 at 50 mg/ml; p < 0.001 and p < 0.001, respectively) (Figure 2B). These data demonstrate that neonatal infection with RSV results in the development of chronic AHR, and furthermore, that airway function is further diminished due to an allergic phenotype.

### Neonatal RSV infection predisposes mice to chronic pulmonary inflammation indicated in BALF cellularity

**Figure 3:**
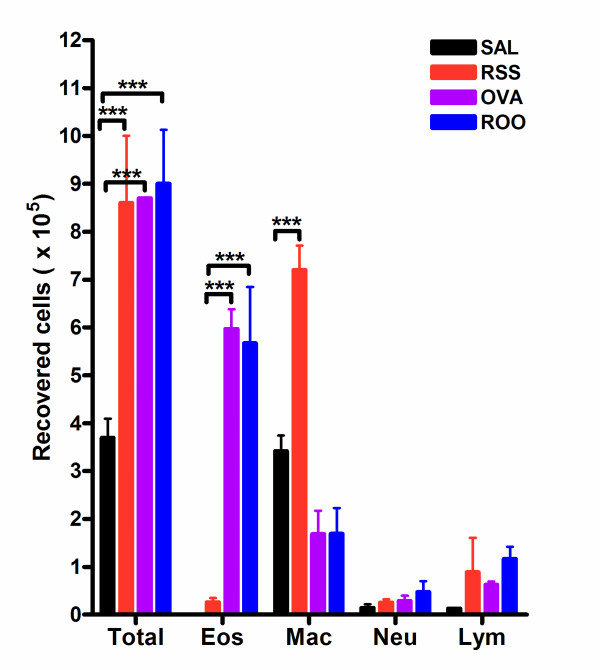
**BAL cellularity in RSV and/or Ova exposed mice**. Bronchoalveolar lavage fluid was isolated on protocol day 69. RSV and/or Ova induced significant increase in total BAL cellularity. In mice exposed to RSV as neonates (RSS), this increase correlated with elevated levels of macrophages; whereas, in mice exposed to Ova (ROO and Ova), this increase correlated to elevation in eosinophil numbers. Data are expressed as means ± SEM, n = 3/group. ***p < 0.001

To evaluate the pulmonary immune response to RSV, BAL fluid was isolated, total cells recovered were counted, and the cellular composition of BALF was determined using morphological criteria. RSV and/or Ova exposed mice recruited significantly more leukocytes than control mice (SAL group, p < 0.001) (Figure 3). However, there was no significant difference in the total number of recovered cells among the RSS, OVA, and ROO groups. Interestingly, BALF cellularity was elevated in mice exposed to RSV as neonates (RSS) on protocol day 69. Upon allergen sensitization and challenge, total BALF cellularity was not significantly altered compared to RSS although the cell populations were changed. Neonatal RSV infection resulted in a predominantly monocyte/macrophage pulmonary infiltrate and these cells seemed to account for the elevation in the total number of recovered cells. In contrast, Ova exposure, in the absence (OVA) or presence of neonatal RSV (ROO) infection, resulted in a significant BALF eosinophilia (compared to control SAL group, p < 0.001). Concomitant with the observed increase in BALF eosinophil numbers in the OVA and ROO groups was a decrease in BALF macrophage numbers.

### Cytokine levels in BAL fluid is altered in mice exposed to RSV and/or Ova

**Figure 4:**
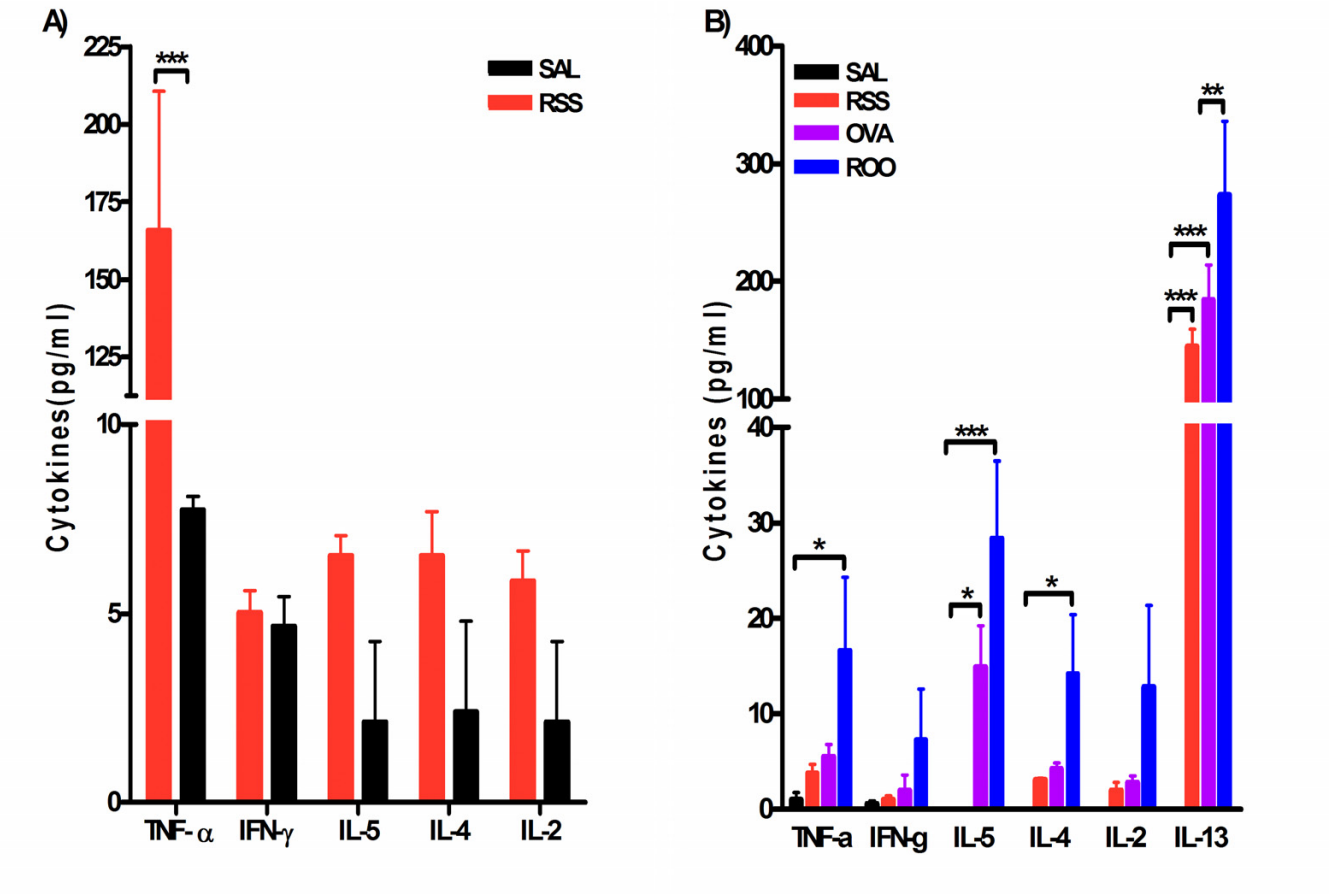
**Cytokine levels in the BAL fluid of mice exposed to RSV and/or Ova**. Bronchoalveolar lavage fluid was isolated 5 hr post-infection with RSV (A) or on protocol day 69 (B). **A**. Elevated levels of TNF-α, IL-5, IL-4, and IL-2 were observed as early as 5 hrs post-infection, although significance over SAL controls was observed only for TNF-α. IL-2, IL-4 and IL-5 were below the limit of detection in the SAL mice. **B**. IL-13 was significantly elevated in RSS mice; while TNF-α, IL-5, and IL-13 were significantly enhanced in mice exposed to RSV and Ova (ROO). IL-13 was below the limit of detection in control animals (SAL). Data are expressed as means ± SEM, n = 3/group. ***p < 0.001, **p < 0.01, and *p < 0.05.

Cytokine levels in BAL fluid were examined to study the Th1/Th2 polarization in RSV and/or Ova exposed mice. As shown in Figure 4A, we assayed cytokine levels in BALF five hours post-infection, and observed greatly elevated TNF-α (21.4 times, p < 0.001) in RSV infected neonates. In addition, Th2 cytokines (IL-4 and IL-5) along with IL-2 were detected in BALF of neonatal infected mice whereas no IL-4, IL-5, or IL-2 was detected in control mice (SAL group). TNF-α levels declined by protocol day 69 in mice infected with RSV as neonates, but was elevated in RSV infected and Ova exposed mice (ROO group, Figure 4B). Intriguingly, IL-13 levels were elevated in RSS mice at protocol day 69 compared to SAL mice (p < 0.001). Mice exposed to RSV and Ova (ROO group) showed increased Th2 cytokines (IL-5 and IL-13) compared to SAL group. In addition, IL-13 levels in ROO mice were significantly elevated compared to the animals exposed to Ova alone (OVA group; p < 0.01). Neonatal infection with RSV also resulted in increased levels of TGF-β_1 _protein in the lungs 4d post-infection (RSS: 29.8 ± 0.45 ng/g lung tissue) compared to mice exposed to Sal alone (22.1 ± 0.39 ng/g lung tissue).

### Enhanced pulmonary histopathology in mice exposed to RSV and/or Ova

**Figure 5:**
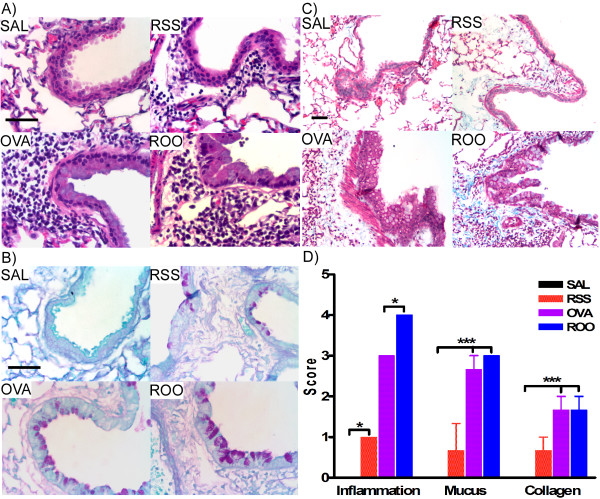
**Lung histopathology of mice exposed to RSV and/or Ova**. Lung tissue sections were obtained from mice on protocol day 69. Tissue sections were stained with H&E (A), PAS (B), and MT (C). **A**. Lung inflammation, **B**. mucus (purple) hyperproduction, and **C**. collagen (blue) deposition were observed in mice exposed to RSV and/or Ova. **D**. Scores were assigned to these histological endpoints by two independent observers and were recorded on a scale of 0–4. Increased deposition of peribronchial and perivascular collagen was observed in the subepithelial reticular layer of the airways in RSS, OVA and ROO mice. In all cases, neonatal RSV infection (RSS) induced persistent lung pathologies including increased peribronchial inflammation, mucus production, and subepithelial fibrosis were exacerbated by subsequent exposure to allergen (ROO). n = 3/group. ***p < 0.001, *p < 0.05.

Airway inflammation, mucus hyperproduction, and collagen deposition in the subepithelial reticular layer of the airway were observed in mice exposed to RSV and/or Ova (Figure 5A: inflammation; Figure 5B: mucus production; Figure 5C: collagen deposition). Mice exposed to both RSV and Ova demonstrated the largest amount of inflammation, mucus production, and collagen deposition (Figure 5D), and this was consistent with the greatest lung resistance and airway hypersensitivity (Figure 2). In addition, tremendous perivascular inflammation was observed in both groups of mice exposed to Ova (i.e., OVA and ROO mice; Figure 5A). Mice infected with RSV as neonates displayed chronic (69d post infection) inflammation, mucus production and collagen deposition compared to control mice (SAL group), which also correlated to enhanced lung resistance and airway hypersensitivity in the RSS group (Figure 2). Increased peribronchial and perivascular collagen deposition and peribronchial smooth muscle deposition indicated that airway remodeling occurred in mice exposed to RSV and/or Ova (Figure 5B), and that neonatal RSV infection alone was sufficient to induce airway remodeling.

## Discussion

In the present study, we have shown that neonatal exposure to RSV alone induced long-term airway hyperresponsiveness and pulmonary resistance in mice. This result was correlated with peribronchial inflammation, increased BALF cellularity, increased mucus production, and airway remodeling. Elevations in TNF-α and Th2 cytokines (IL-4 and IL-5) were observed in BALF immediately following neonatal RSV infection. Once these mice had matured (protocol day 69), only IL-13 remained elevated in the BALF. Neonatal RSV infection followed by adult exposure to allergen resulted in significantly higher lung resistance, along with increased total cellularity, eosinophilia, and increased TNF-α and Th2 cytokines (IL-5 and IL-13) in BALF. In addition, TNF-α and IL-13 were significantly higher compared to mice exposed to Ova alone. The most severe lung histopathology was observed in mice exposed to both RSV and Ova, as indicated by severe peribronchial and perivascular inflammation, mucus production, and collagen deposition. Collectively, these data suggest that neonatal RSV infection influences adult immune response to allergen (Ova) and exacerbates allergic pathophysiology in mice long after viral titers are no longer detectable.

In contrast to previously published studies analyzing the effect of RSV infection on allergen exposure in adult mice. [17], the present study investigated the influence of neonatal RSV infection on adult allergen sensitization. Neonatal infection with RSV alone was sufficient to induce long-term pulmonary dysfunction and inflammation. Similarly, neonatal infection of brown Norway rats with parainfluenza type 1 (Sendai) virus also led to increases in pulmonary resistance and hyperresponsiveness to methacholine up to 65d after infection [18]. As in our model, persistent airway dysfunction following neonatal infection with Sendai virus correlated with increased peribronchial fibrosis and pulmonary inflammation. [19]. Furthermore, both models (i.e., neonatal RSV infection and neonatal Sendai virus infection) resulted in significantly increased mRNA [19] and protein levels for TGF-β_1_. Although numerous cytokines may contribute to airway remodeling, the elevation of TGF-β_1_, a fibrogenic cytokine, in both neonatal viral infection models prior to the development of fibrosis suggests a role this cytokine in the regulation of viral-induced airway remodeling observed in neonates.

More importantly, neonatal infection with RSV predisposed mice to the development of enhanced AHR and inflammation after allergen exposure. In contrast, adult RSV infection prior to allergen exposure seemed to assert a "protective" response as evidenced by significantly decreased allergen induced pulmonary resistance, tissue eosinophilia, and IL-13 levels [20]. Our data presented here and elsewhere [16] extend these findings and, more importantly, demonstrate that the age at initial RSV infection also determines whether RSV infection will exacerbate or prevent subsequent allergic inflammation.

A more recent study using neonatal RSV infection followed by subsequent reinfection of adults with RSV demonstrated that early RSV infection also exacerbates RSV induced diseases in the adult [13]. Interestingly, if the primary RSV infection occurred at 3 wks of age, a protective effect upon secondary infection was observed similar to that reported by Peebles and colleagues [17]. Dakhama and colleagues further established that enhancement of AHR, pulmonary eosinophilia, and mucus hyperproduction during reinfection were dependent on IL-13 [13]. We demonstrated that neonatal RSV infection alone leads to elevated levels of IL-13 in the lung and that exposure to allergen significantly increases IL-13 levels over RSV exposure alone. IL-13 has emerged as a major regulatory molecule involved in mucus hyperproduction and allergen induced AHR [21-23]. It is entirely possible that the long-term AHR, pulmonary inflammation, and mucus production observed in our neonatal RSV model is due to high levels of IL-13. In fact, elevated levels of IL-13 were observed in whole lung homogenates as early as 5 hours post-infection (data not shown) and were again observed in adult lungs on protocol day 69 suggesting that IL-13 is being chronically produced. Although the exact cellular source of IL-13 in this neonatal model of RSV infection is currently unknown, previous studies have demonstrated that epithelial cells and/or macrophages infected by RSV are a significant source of IL-13 and are capable of producing this cytokine for months after the initial infection. [24]. We are currently investigating this possibility.

In our study, TNF-α was significantly elevated in the BALF shortly after infection with RSV. TNF-α is an important cytokine for innate immune responses and a central regulator of inflammatory processes, through binding to distinct membrane receptors, referred to as p55 or TNFR1 and p75 or TNFR2 [25]. TNF-α is likely a central mediator of airway inflammation and AHR in asthma, regulating inflammatory cell infiltration, locally enhancing vascular permeability, and inducing the release of the chemokines. Ultimately, this will lead to chronic inflammation and irreversible airway remodeling. Recently, depletion studies using monoclonal antibody therapy for TNF-α have shown promising effects in viral-specific lung immunopathology. [26], rheumatoid arthritis [27] and inflammatory bowel disease [28]. Moreover, a soluble TNF receptor fusion protein, etanercept, has proven efficacious in treating juvenile rheumatoid arthritis in patients as young as 4 [27]. In viral models, TNF-α depletion reduced recruitment of inflammatory cells, reduced type 1 and type 2 cytokines in BALF, and decreased pulmonary pathology without inhibiting viral clearance [26,29]. Although the precise mechanism by which TNF-α leads to the pathology in lungs after RSV infection remains unknown, our data and these previous studies suggest a key role for TNF-α in chronic inflammation in the lung and subsequent airway remodeling associated with asthma.

Several studies along with our present data have established the correlation of severe RSV infection followed by allergen exposure and the development of allergic inflammatory disease (i.e., asthma) in mice. Although the mechanism by which the exposure causes asthma and the importance of such exposures in humans need to be further elucidated, our current and previously published data [16] demonstrate that the initial age of the RSV infection is capable of altering adult pulmonary function and exacerbating pulmonary immunopathology when followed by subsequent allergen exposure. Furthermore, enhanced AHR correlated with chronic pulmonary inflammation, upregulation of the Th2 cytokine, IL-13, and subepithelial fibrosis of the bronchial airways. Increases in TNF-α within 5 hours of RSV infection in our mouse model also suggest a role for this cytokine in the immunopathology of RSV-induced wheeze and asthma development in humans.

## Conclusion

We have demonstrated that neonatal infection with RSV in mice leads to reduced lung function, which is associated with chronic inflammation, increased mucus production, and airway remodeling in the lung. In addition, RSV infection in neonates predisposes the adult to develop enhanced airway responses upon allergen exposure. The upregulation of TNF-α and IL-13 suggests that these cytokines may play a key role in mediating this process and highlights the importance of these cytokines as therapeutic targets for RSV induced asthma.

## Abbreviations

RSV, respiratory syncytial virus; AHR, airway hyperreactivity; Ova, ovalbumin; Sal, saline; BAL, bronchoalveolar lavage; BALF, bronchoalveolar lavage fluid; MeCh, methacholine; Penh, enhanced pause, TNF-α: Tumor necrosis factor-α

## Competing interests

The author(s) declare that they have no competing interests.

## Authors' contributions

DY performed pulmonary function tests, cytokine assays, and assisted in the preparation of the manuscript. DB counted the BAL fluid differentials and assisted in the preparation of the manuscript. KW counted the BAL fluid differentials and assisted in the preparation of the manuscript and the micrographs. MR determined pulmonary viral titers. MD helped in preparation of reagents. SC conceived of the study, performed/assisted in all experiments, and prepared the manuscript. All authors read and approved the final manuscript.
